# Thirty-day incidence of stroke after transfemoral transcatheter aortic valve implantation: meta-analysis and mixt-treatment comparison of self-expandable versus balloon-expandable valve prostheses

**DOI:** 10.1007/s00392-020-01775-x

**Published:** 2020-11-29

**Authors:** Philipp C. Seppelt, Silvia Mas-Peiro, Roberta De Rosa, Zisis Dimitriasis, Andreas M. Zeiher, Mariuca Vasa-Nicotera

**Affiliations:** 1 Division of Cardiology, Department of Medicine III, University Hospital, Goethe University Frankfurt, Frankfurt am Main, Germany; 2 DZHK partner site Rhine-Main, German Centre for Cardiovascular Research, Berlin, Germany

**Keywords:** Aortic stenosis, Stroke, Balloon-expandable TAVI, Self-expandable TAVI

## Abstract

**Aims:**

Stroke is a major complication after transcatheter aortic valve implantation (TAVI). Although multifactorial, it remains unknown whether the valve deployment system itself has an impact on the incidence of early stroke. We performed a meta- and network analysis to investigate the 30-day stroke incidence of self-expandable (SEV) and balloon-expandable (BEV) valves after transfemoral TAVI.

**Methods and results:**

Overall, 2723 articles were searched directly comparing the performance of SEV and BEV after transfemoral TAVI, from which 9 were included (3086 patients). Random effects models were used for meta- and network meta-analysis based on a frequentist framework. Thirty-day incidence of stroke was 1.8% in SEV and 3.1% in BEV (risk ratio of 0.62, 95% confidence interval (CI) 0.49–0.80, *p* = 0.004). Treatment ranking based on network analysis (P-score) revealed CoreValve with the best performance for 30-day stroke incidence (75.2%), whereas SAPIEN had the worst (19.0%). However, network analysis showed no inferiority of SAPIEN compared with CoreValve (odds ratio 2.24, 95% CI 0.70–7.2).

**Conclusion:**

Our analysis indicates higher 30-day stroke incidence after transfemoral TAVI with BEV compared to SEV. We could not find evidence for superiority of a specific valve system. More randomized controlled trials with head-to-head comparison of SEV and BEV are needed to address this open question.

**Graphic abstract:**

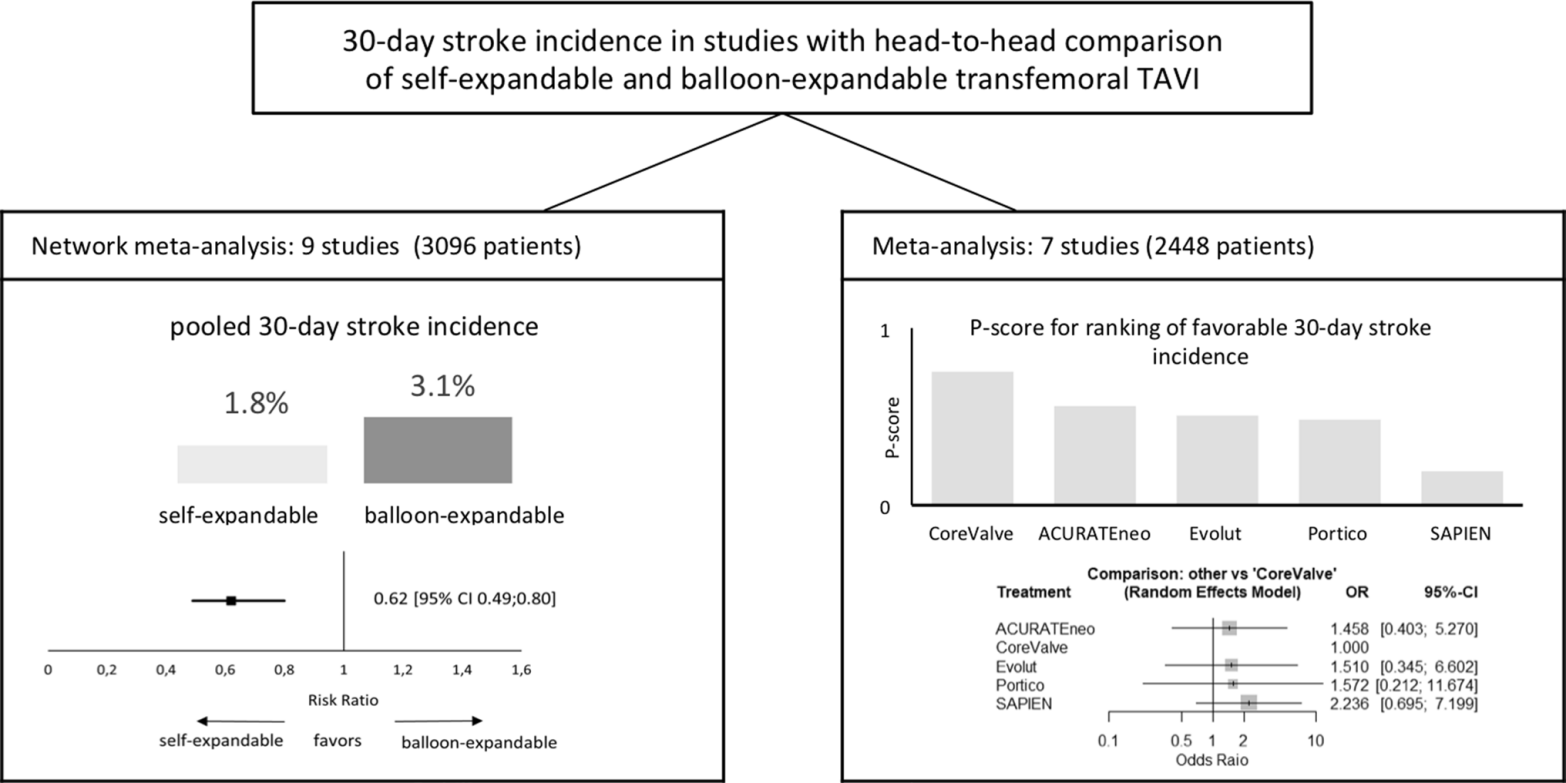

## Introduction

Since the first-in-man transcatheter aortic valve implantation (TAVI) performed in Rouen in 2002, TAVI has radically transformed the treatment of aortic valve stenosis [1]. Nowadays, the indication for interventional aortic valve replacement is expanded from patients with high to intermediate perioperative risks [2, 3]. The recently published PARTNER 3 trial even testified a superior overall outcome, regarding a composite endpoint of death, stroke or rehospitalization, in patients with aortic valve stenosis and low perioperative risk undergoing TAVI compared to surgical aortic valve replacement (SAVR) [4]. Currently, several approved valve deployment systems from different manufactures are on the market and simplified, most can be divided into self-expandable (SEV) and balloon-expandable (BEV) valves.

Stroke is a major and one of the most feared complications after transcatheter aortic valve implantation (TAVI) and part of the updated endpoint definitions established by the valve academic research consortium (VARC-2) [5]. Stroke, in general, is a major limitation for quality of life and a cost-effective complication [6, 7].

A large meta-analysis by Shah et al. from 2018 determined a periinterventional risk of stroke within the first 30 days of 2.7% after TAVI [8]. Stroke within the early phase post TAVI occurs likely implantation and devices related, whereas stroke occurring later is regarded to be associated with individual patient’s risk factors and long-term valve performance [9].

However, so far it remains uncertain, whether self- or balloon-expandable valves perform differently concerning 30-day stroke incidence. To investigate, whether SEV or BEV has the greater risk for stroke within the first 30 days after procedure with a transfemoral approach, we performed a meta- and network meta-analysis of the recent literature.

## Methods

**Fig. 1:**
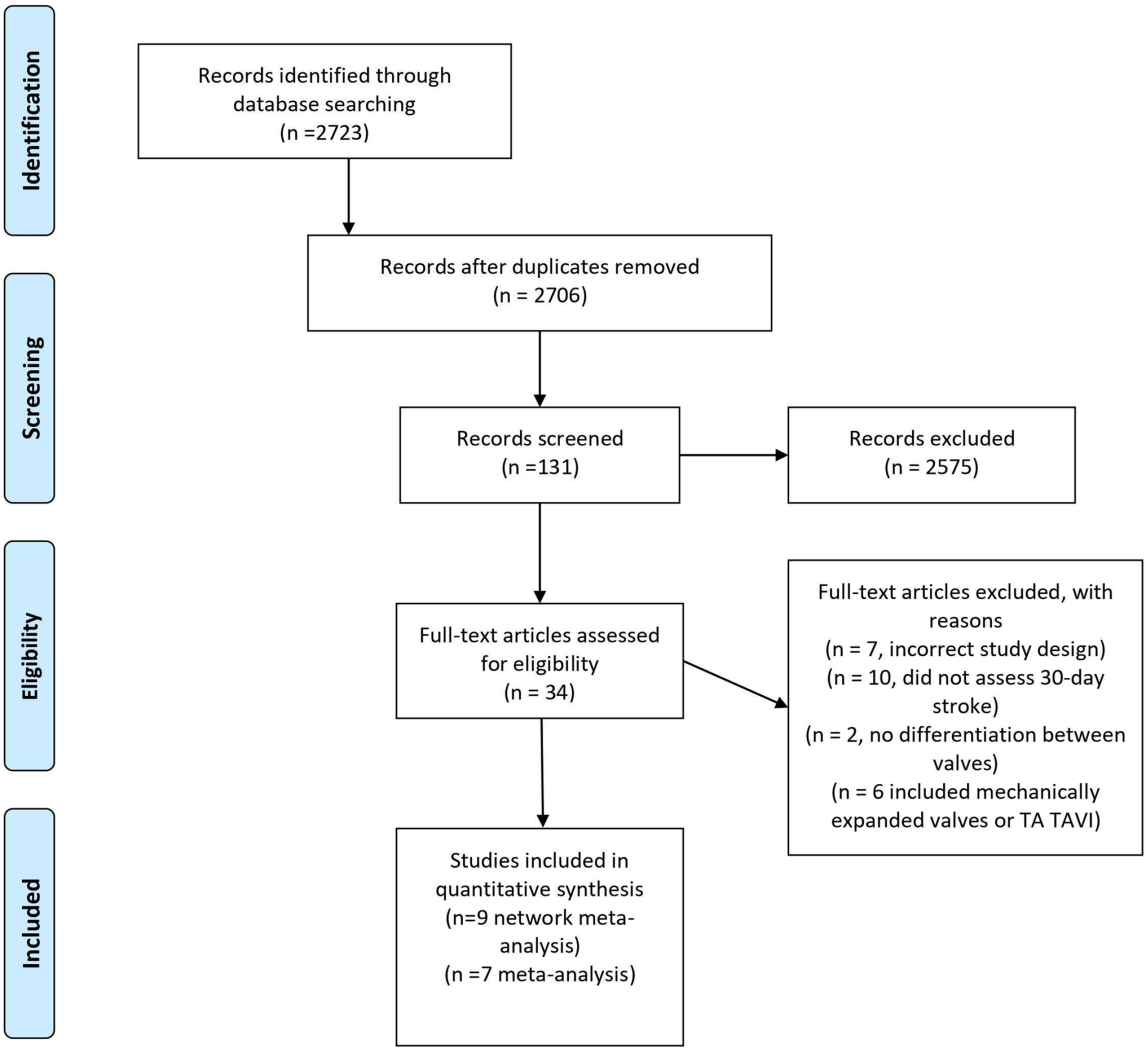
PRISMA search protocol. Data bases screened: MEDLINE/Pubmed, ClinicalTrials.gov and Cochrane Library. Detailed search protocol with applied search terms can be found in Supplements. For meta-analysis, only studies with head-to-head comparison of SEV and BEV were included, whereas for network meta-analysis also studies comparing different SEV were considered. *BEV* balloon-expandable valve, *TA* transapical, *TAVI* transcatheter aortic valve implantation, *SEV* self-expandable valve

The primary outcome of interest was the 30-day incidence of postoperative stroke after transfemoral TAVI with BEV and SEV. Randomized controlled and propensity pair-matched studies with at least 40 patients per group published in English from the time of the first TAVI procedure in 2002 until November 29, 2019 were included. Only studies reporting the 30-day stroke incidence in a head-to-head comparison of SEV and BEV were included, whereas studies comparing TAVI with surgical aortic valve replacement were excluded from analysis. Furthermore, studies or registries not reporting the outcome of transfemoral and transapical TAVI separately were not considered for analysis. Only studies reporting the procedural outcome according to the standardized VARC-2 criteria (or first VARC criteria for older studies) were included [5, 10]. According to VARC-2 criteria, periprocedural stroke is determined clinically or by neuroimaging. Diagnosis must be confirmed by a neurologist or neurosurgical specialist or by a neuroimaging procedure, but stroke may be diagnosed on clinical grounds alone. For network meta-analysis, studies comparing different SEV valves in a head-to-head comparison were also included. Because of inconsistency in direct and indirect estimates, trials comparing SAVR with BEV or SEV, such as the PARTNER trial series and the SURTAVI trial, were not considered for network meta-analysis [9, 11–14]. Three databases, MEDLINE/Pubmed, ClinicalTrials.gov and Cochrane Library, were searched applying predefined key search terms that are presented in the Supplements. Studies were screened at title/abstract level by two independent reviewers (PCS and SMP). Studies matching the inclusion criteria were analyzed at full text level and the quality of evidence was assessed keeping with the Joanna Briggs Institute (JBI) Critical Appraisal Checklist for Studies Reporting Prevalence Data (Supplement Table 1) and Preferred Items for Reporting of Systematic Reviews and Meta-analyses (PRISMA) [15, 16]. Disagreements between the reviewers were resolved through discussion. Meta- and network meta-analysis were carried out using statistical analysis software R (Version 3.6.1, “meta” and “netmeta” package, r-project.org). For meta-analysis, more conservatively random effects models were used due to heterogeneity in study methodology and population and risk ratios were calculated (RRs). Heterogeneity and among study variance were assessed by calculating Cochran’s Q, I^2^ and *τ*^2^ (Sidik–Jonkman estimator) [17]. Specifically, I-squared >50% was considered evidence of moderate or severe inconsistency. To compare different types of valves, a random effects network meta-analysis based on a frequentist framework for indirect and mixed comparisons was applied and reported odds ratios (ORs). First, we generated a comparison-adjusted funnel plot to assess potential publication bias and plot asymmetry was evaluated by Egger’s Test [18] (Supplements, Fig. 1). Heterogeneity and among study variance were estimated as described above. Additionally, net heat and net splitting were generated to determine study network inconsistency. Using P-Score, the relative ranking probability of each valve was estimated, and the hierarchy of competing valves was obtained. Shortly, P-scores estimate the probability for each treatment of being better than the competing treatments. However, P-score ranking probability does not correlate with relative treatment effects and cannot be interpreted clinically.

**Table 1:** Study and patient characteristics

Study	Design	Valves	*n* =	Age (years)	Sex (female)	Operative risk	Previous stroke	30-day stroke	Annotations; applied criteria for stroke	Analysis
Abdel-Wahab et al. 2014	RCT (CHOICE Trial)	CoreValve (SE)	117	79.6	71.7%	Intermediate	–	2.6,%	VARC	Meta- and network meta-analysis
		Sapien XT (BE)	121	81.9	57%	Intermediate	–	5.8%		
Zhang et al. 2015	PSM	CoreValve (SE)	40	81.3	50%	High	–	0%	VARC-2	Meta- and network meta-analysis
		Sapien 3 (BE)	40	82.1	52.5%	High	–	2.5%		
Landes et al. 2017	PSM	CoreValve (SE)	73	82	70%	Intermediate	16%	1.4%	VARC-2	Network meta-analysis
		Evolut R (SE)	73	82	73%	Intermediate	22%	1.4%		
Husser et al. 2017	PSM	ACURATEneo (SE)	311	81	60.8%	Intermediate	13.8%	2.3%	VARC-2	Meta- and network meta-analysis
		Sapien 3 (BE)	622	81	55.3%	Intermediate	12.5%	3.1%		
Enriquez-Rodriguez et al. 2018	PSM	Evolut R	64	84	58%	Intermediate	–	0%	VARC-2	Meta- and network meta-analysis
		Sapien 3 (BE)	80	83	55%	Low	13%^§^	3%		
Lanz et al. 2019	RCT	ACURATEneo (SE)	367	82.6	59%	Low	13%^§^	2%	^§^Stroke and transient ischemic attack combined reported; VARC-2	Meta- and network meta-analysis
		Sapien 3 (BE)	364	83	55%	Low	13%^§^	3%		
Mas et al. 2019	PSM	Portico (SE)	104	81.8	41.3%	Low	14.4%	2.9%	VARC-2	Meta- and network meta-analysis
		Sapien 3 (BE)	73	81.5	34.2%	Low	17.8%	4.1%		
Pagnesi et al. 2019	PSM	ACURATEneo (SE)	251	81.4	65.7%	Intermediate	10%	2.4%	VARC-2	Network meta-analysis
		Evolut Pro (SE)	251	81.6	65.7%	Intermediate	7.9%	2.8%		
Costa et al. 2020	PSM	Sapien 3 (BE)	48	83	68.8%	Low	4.2%	0%	VARC-2	Meta- and network meta-analysis
		Evolut R (SE)	48	83	68.8%	Low	6.3%	0%		
		ACURATEneo (SE)	48	82	70.8%	Intermediate	2.1%	0%		

Operative risk according to STS SCORE; if not specified, logistic EuroSCORE II or EuroSCORE was used for risk stratification. For more detailed patient characteristics please, see Supplement Table 3

*BE* balloon-expandable, *PSM* propensity score matching, *RCT* randomized controlled trial, *SE* self-expandable, *VARC-2* Valve Academic Research Consortium

## Results

**Fig. 2:**
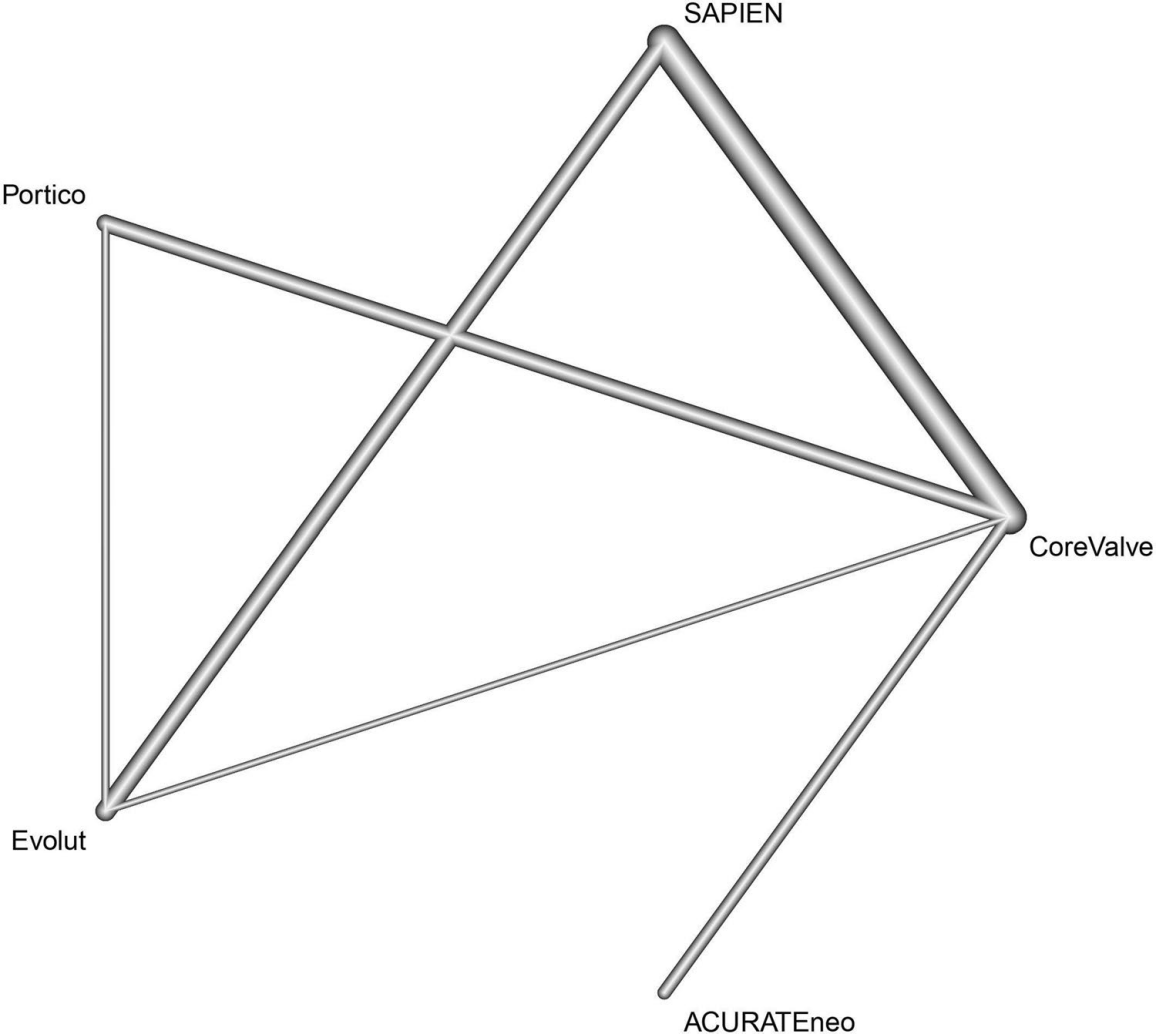
Study network. Study network displaying direct (connection) and indirect evidence (over network link). Thickness of lines corresponds to number of specific comparisons

Overall, a total of 2723 citations were initially retrieved and 9 studies were finally included according to pre-specified criteria, with a total of 3096 patients (1351 patients receiving SEV and 1745 patients receiving BEV (Table 1, Figs. 1, 2). Out of these nine selected studies, only two had randomized controlled character (Abdel-Wahab et al. 2014 and Lanz et al. 2019) [19, 20], whereas in seven patient propensity score matching was conducted to adjust for baseline characteristics. For meta-analysis, seven studies comparing head-to-head SEV and BEV were included [19–25], whereas for network analysis all nine studies were considered, including two studies comparing different SEV [26, 27]. Five studies reported the outcome of patients with intermediate, two with low, one with low to intermediate and one with high operative risk according to the operative risk models STS score (The Society Thoracic of Surgeons Score, Table 1) [28]. If STS score was not specified, EuroSCORE and EuroSCORE II were used for risk classification (European System for Cardiac Operative Risk Evaluation) [29, 30]. Thirty-day stroke incidence varied in procedures with SEV from 0% (CoreValve, Evolute R, ACURATEneo) to 2.9% (Portico) and in BEV from 0% (SAPIEN 3) to 5.8% (SAPIEN XT, Table 1).

**Fig. 3:**
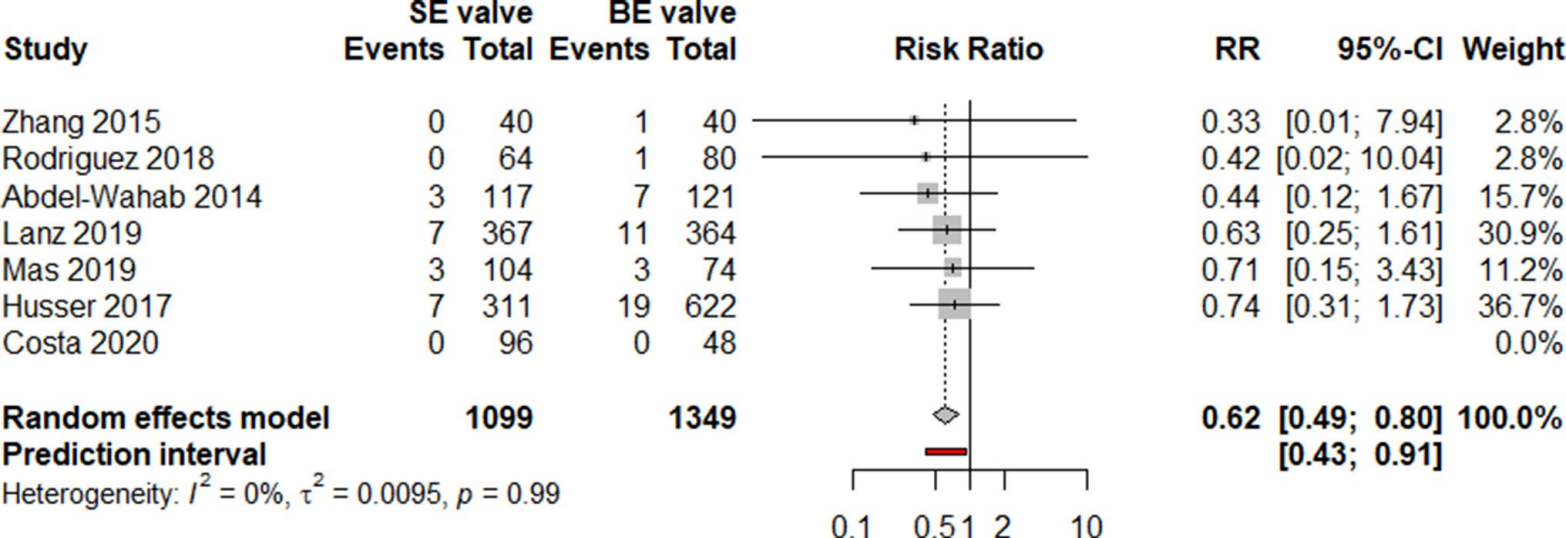
Meta-analysis comparing SEV and BEV concerning incidence of 30-day stroke. Meta-analysis revealed favorable 30-day incidence of stroke after TAVI with SE compared to BE valves (RR 0.62 95%-CI 0.49–0.8, overall *p* = 0.0043; Cochrane’s *Q* = 0.64, *p* = 0.986). *BE* balloon-expandable, *RR* risk ratio, *SE* self-expandable, *TAVI* transcatheter aortic valve implantation

Meta-analysis revealed a pooled thirty-day incidence of stroke of 1.8% in SEV (20 out of 1099) and 3.1% in BEV (42 out of 1349) resulting in a RR of 0.62 [95% confidence interval (CI) 0.49–0.80, *p* value 0.004, Fig. 3] in favor for SEV. Between-study heterogeneity was low, as indicated by *I*^2^ (0%) and *τ*^2^ (0.095, *p* = 0.99). Prediction interval presents the expected range of true effects in similar studies and ranged from 0.43 to 0.91 (Table 2).

**Table 2:** Net league

ACURATEneo	.	0.85 (0.28; 2.58)	.	0.68 (0.36; 1.30)
1.46 (0.40; 5.27)	CoreValve	1.00 (0.06; 16.30)	.	0.41 (0.12; 1.46)
0.97 (0.36; 2.62)	0.66 (0.15; 2.90)	Evolut	.	0.41 (0.02; 10.26)
0.93 (0.16; 5.31)	0.64 (0.09; 4.72)	0.96 (0.13; 6.89)	Portico	0.70 (0.14; 3.58)
0.65 (0.35; 1.22)	0.45 (0.14; 1.44)	0.68 (0.22; 2.05)	0.70 (0.14; 3.58)	SAPIEN

Net league reporting the estimated pooled effect sizes (OR with 95%CI) generated by direct comparisons (upper triangle, gray background) and by combination of direct and indirect comparisons (lower triangle, white background)

**Fig. 4:**
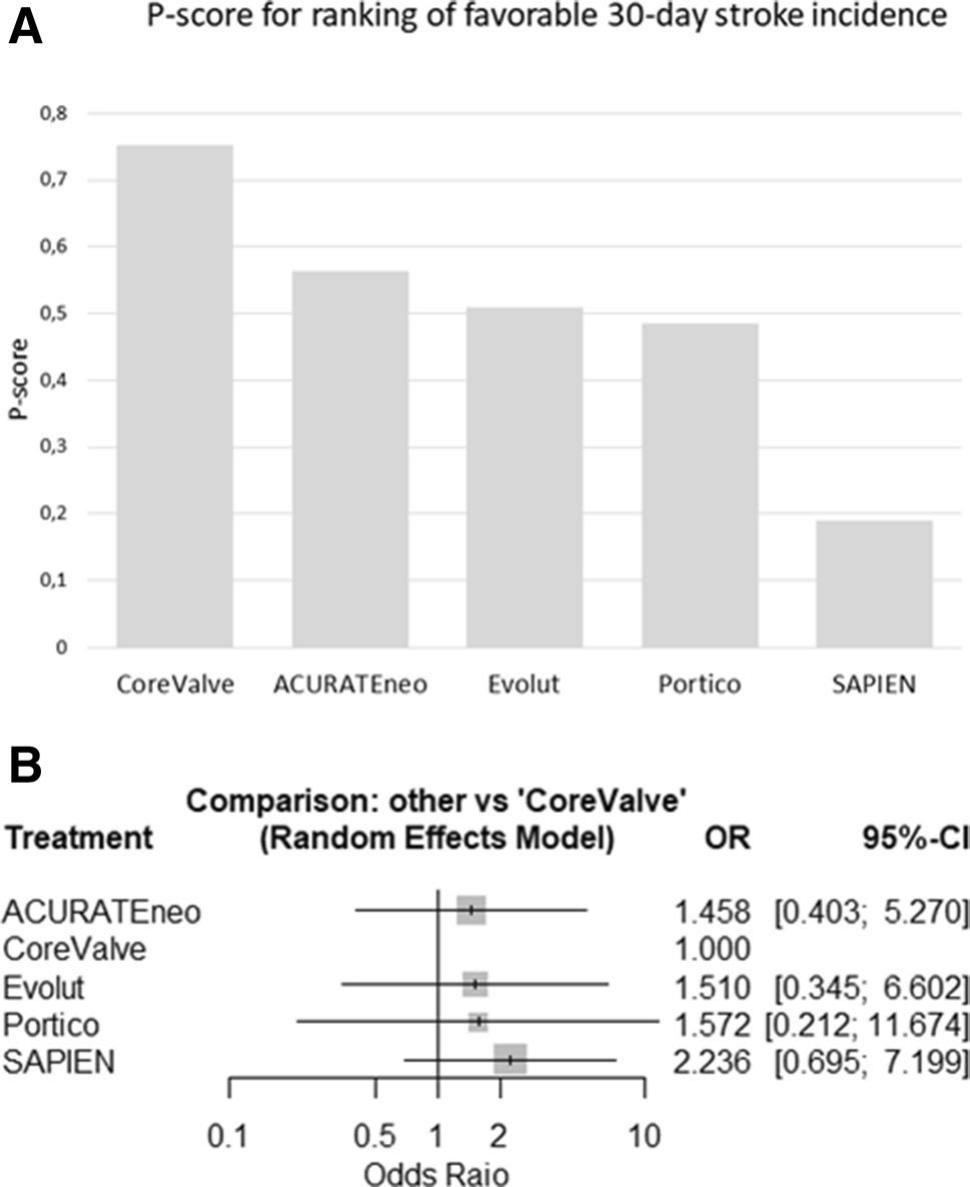
30-day stroke incidence in competitive valves: **a** According to P-score derived from random effect frequentist analysis, CoreValve showed the best performance regarding incidence of 30-day stroke. **b** Pooled analysis demonstrated no superior treatment effect of self-expandable CoreValve concerning 30-day incidence of stroke compared to other self-expandable or balloon-expandable valves (Cochrane’s *Q* = 0.34, *p* = 0.987, *I*^2^ = 0% and *τ*^2^ = 0%). *OR* odds ratio

Nine studies were included for network meta-analysis addressing head-to head comparison of SEV with BEV by combining direct and indirect comparisons. Relevant study heterogeneity and inconsistency could be ruled out (Supplement Figs. 1, 2 and 3). In accordance with the estimated P-Score, Medtronic CoreValve was best effective for a reduction of 30-day stroke (75.2%, pooled stroke incidence 1.7%), whereas the worst were SAPIEN 3 and XT (19.0%, pooled stroke incidence 3.1% Fig. 4a). However, combined direct and indirect evidence showed no inferiority of SAPIEN valves in head-to-head comparison with CoreValve (OR 2.24, 95% CI 0.70–1.7.20) concerning 30-day stroke incidence (Fig. 4b).

## Discussion

Stroke after transfemoral TAVI is a potentially life-threatening and quality of life-impacting event. Although the incidence of stroke following TAVI declines continuously in recent years, it remains a significant cause of mortality and morbidity. The question, whether SEV or BEV has different early-phase stroke rates post TAVI, has been not sufficiently addressed yet.

Our analysis reveals some interesting findings. First, we found a significantly reduced 30-day incidence of stroke after transfemoral TAVI with SEV compared to BEV, resulting in a relative risk reduction of 38% in favor for SEV. Overall, 30-day stroke incidence was low after TAVI in both SEV (1.8%) as well as after BEV (3.1%) and varied significantly between the different included studies. Different to our results, a meta-analysis by Agarwal et al. reported a similar 30-day stroke incidence for transfemoral TAVI with BEV and SEV of 2.6% [31]. Important to note, different to our analysis, this study included also multicenter registries. Moreover, distribution analysis of the pooled estimates indicated significant heterogeneity.

Second, our mixt-treatment analysis revealed the best performance concerning 30-day stroke for self-expandable CoreValve. We calculated a pooled 30-day stroke incidence for CoreValve of 1.7%, remarkably superior to the stroke rate of the randomized controlled COREVALVE trial, comparing TAVI versus SAVR, that reported a risk of disabling stroke at 30-day follow-up of 3.9% for TAVI with CoreValve in year 2014. Our meta-analysis revealed for SAPIEN valves the worst performance of all investigated valves (pooled 30-day stroke incidence 3.1%). Contrary to our findings, the consecutive randomized controlled PARTNER trials recorded significantly improved stroke rates over the years. The most recent PARTNER 3 trial set a new remarkable benchmark for TAVI procedures and reported a 30-day stroke rate of 0.6% with the balloon-expandable SAPIEN 3. However, it is important to note that this was observed in patients with low operative risk as indicated by a median STS score of 1.9% [32]. Nevertheless, due to the different study design comparing TAVI with SAVR or best medical therapy, the PARTNER trials with remarkable outcome results, especially of the recent one, were not included in our study.

Third, CoreValve was the best effective of the investigated valves to reduce 30-day stroke rate. Nonetheless, in head-to-head comparison we could not proof superiority of one valve over another. A network meta-analysis by Biondi-Zoccai et al. compared the outcome of TAVI vs. SAVR and included four RCT with different follow-up periods (CHOICE trial, PARTNER Cohort A trial, STACCATO trial, US CoreValve trial) [33]. In a sub-analysis, CoreValve demonstrated lower stroke incidence after transfemoral TAVI than SAPIEN valves (OR  0.32, 95% CI 0.13–0.73) after a median follow-up of 8 months (ranging from 1 to 12 months). Comparatively, a pooled analysis of 5097 patients by Eggebrecht et al. reported a higher stroke rate after TAVI with SAPIEN compared to CoreValve (3.5% vs. 1.5%), whereas another meta-analysis by Athappan et al. differentiated between multi- and single-center experience and suggested similar risk of 30-day stroke for CoreValve and SAPIEN valves (OR 1.10, 95% CI 0.79–1.15 and OR 1.28, 95% CI 0.43–3.81, respectively) [34]. As the TAVI technology and learning curves continue to advance, randomized controlled trials comparing the newer, improved SEV and BEV are needed to finally address this open question directly.

What are the pathophysiological mechanisms of stroke during the early phase of TAVI procedures? Plausible causes of brain injury during TAVI are dysregulated cerebral perfusion, such as hypoperfusion or hypertension, hemorrhagic complications with potential relation to anticoagulation and emboli [35]. Manipulation of the aortic root and aortic arch and navigation of catheters and wires across the aortic valve can provoke embolization. Implantation of both SEV and BEV can cause embolization but highly likely with differences in timing. Kahlert et al. performed transcranial Doppler examinations during TAVI and reported that risk of embolization with SEV valves is increased during slow stepwise implantation and with BEV during valve positioning prior to final implantation [36]. Implantation of BEV requires rapid ventricular pacing for stabilization of the aortic root and accurate positioning of the prosthesis. Rapid ventricular pacing causes a state of low to no cardiac output resulting in cerebral hypoperfusion and may be associated with increased stroke risk [37, 38]. Balloon post-dilatation is routinely applied with SEV and BEV to reduce paravalvular leak and has been described as predictor for acute cerebrovascular events [39].

Although we made efforts to restrict limitations typical for all meta-analyses and mixed treatment comparisons by addressing Preferred Reporting Items for Systematic Reviews and Meta-analyses guidelines, determining source of heterogeneity and publication bias, this study has unavoidable limitations. Although we only included randomized controlled trials and propensity score matched studies addressing a direct comparison of SEV and BEV, the effects of different baseline characteristics on stroke incidence cannot be fully assessed. The median operative risk, assessed by EuroScore and STS score, was heterogenous in the included studies. Several randomized controlled TAVI trials compared TAVI vs. SAVR, such as the PARTNER trial series and the SURTAVI trial [9, 11–14]. Due to our chosen inclusion criteria and because of inconsistency in direct and indirect estimates, trials comparing SAVR with BEV or SEV were not considered for our analysis. Furthermore, we noted a variability in the definition and specification of stroke. Only trials reporting stroke incidence according to the VARC criteria were included. Seven trials reported outcomes according to the recent VARC-2 criteria and one trial according to the first and older VARC criteria published in 2011 [5, 10]. Nevertheless, the included studies did not report in detail how stroke was diagnosed, either by neurological assessment or neuroimaging procedures, a circumstance that arises possible ascertainment bias.

## Conclusion

Stroke after TAVI remains a pivotal clinical complication significantly impacting mortality and morbidity. Our meta-analysis indicates a higher 30-day incidence of stroke after transfemoral TAVI with BEV compared to SEV. Furthermore, we could not find evidence for superiority of a specific valve system. There is a need for more randomized controlled trials with head-to-head comparison of SE and BE valves to address this open question.

## Data Availability

Upon request.

## Abbreviations

BE: Balloon-expandable
BEV: Balloon-expandable valve
CI: Confidence interval
EuroSCORE: European system for cardiac operative risk evaluation
JBI: Joanna Briggs institute critical appraisal checklist for studies reporting prevalence data
PRISMA: Preferred items for reporting of systematic reviews and meta-analyses
RR: Risk ratio
OR: Odds ratio
SAVR: Surgical aortic valve replacement
SE: Self-expandable
SEV: Self-expandable valve
STS score: The society thoracic of surgeons score
TAVI: Transcatheter aortic valve implantation
VARC: Valve academic research consortium

## Code availability

Please see “Supplementary material”.
